# S100A7 attenuates immunotherapy by enhancing immunosuppressive tumor microenvironment in lung squamous cell carcinoma

**DOI:** 10.1038/s41392-022-01196-4

**Published:** 2022-10-21

**Authors:** Chengming Liu, Sufei Zheng, Zhiliang Lu, Zhanyu Wang, Sihui Wang, Xiaoli Feng, Yan Wang, Nan Sun, Jie He

**Affiliations:** 1 Department of Thoracic Surgery, National Cancer Center/National Clinical Research Center for Cancer/Cancer Hospital, Chinese Academy of Medical Sciences and Peking Union Medical College, 100021 Beijing, China; 2 Department of Pathology, National Cancer Center/National Clinical Research Center for Cancer/Cancer Hospital, Chinese Academy of Medical Sciences and Peking Union Medical College, 100021 Beijing, China; 3 Department of Medical Oncology, National Cancer Center/National Clinical Research Center for Cancer/Cancer Hospital, Chinese Academy of Medical Sciences and Peking Union Medical College, 100021 Beijing, China

**Keywords:** Cancer therapy, Lung cancer

**Dear Editor**,

In recent years, the rapid development of immunotherapy has brought survival benefits for lung squamous cell carcinoma (LUSC) patients who have rare treatment options. Nevertheless, there are still some patients with immunotherapy resistance, which is the root cause of the low benefit rate and disease progression of the overall population, and it is an urgent problem to be solved. Except for tumor mutation burden, the tumor immune microenvironment (TIME) as characterized by infiltration of CD8^+^ tumor-infiltrating lymphocytes (TILs) and PD-L1 expression has been associated with the efficacy of immune checkpoint inhibitors (ICIs). In our previous studies, we have demonstrated that psoriasin, also known as S100 calcium-binding protein A7 (S100A7), not only accelerates tumor progression via the activation of the p-Erk pathway in LUSC and the p-Erk and p-FAK pathways in esophageal squamous cell carcinomas (ESCC) but also remodels the tumor microenvironment by promoting angiogenesis and M2 macrophage infiltration in ESCC.^1,2^ However, the role of S100A7 in the TIME and immunotherapy efficacy in LUSC remains to be elucidated.

In this study, a cohort of 102 LUSC resected specimens from the Cancer Hospital and Institution, Chinese Academy of Medical Sciences was collected to determine the correlation between S100A7 expression and TIME based on CD8^+^ TILs infiltration and PD-L1 expression using immunohistochemistry. The results showed that S100A7 expression was negatively related with both PD-L1 expression (*r* = −0.25, *p* = 0.010; Fig. 1a) and the CD8^+^ TILs infiltration (*r* = −0.31, *p* = 0.002; Fig. 1b). Several chemokines including CCL5, CXCL9, CXCL10, and CXCL11 have been shown to drive the infiltration of CD8^+^ TILs.^3,4^ Considering this, we investigated the relationship between the expression of S100A7 and these chemokines using transcriptome data of LUSC samples from The Cancer Genome Atlas (TCGA) database. A negative association between S100A7 and CXCL9 expression was observed (*r* = −0.14, *p* = 0.002; Fig. 1c and Supplementary Fig. S1b). Furthermore, we applied CIBERSORT to calculate the individual fractions of 22 immune cells based on transcriptome data of LUSC samples and analyze their relationship with S100A7 expression. In addition to its involvement with CD8^+^ T cells (*r* = −0.21, *p* < 0.001), S100A7 expression also negatively correlated with M1 macrophage infiltration (*r* = −0.17, *p* < 0.001) (Supplementary Fig. S1c). Subsequent immunohistochemistry studies further validated the negative relationship of S100A7 expression with both CXCL9 expression (*r* = −0.45, *p* < 0.001; Fig. 1d) and CD68^+^ M1 macrophages infiltration (*r* = −0.27, *p* = 0.006; Fig. 1e) in LUSC samples. Next, RT-qPCR was performed to measure the expression of M1 macrophage-specific marker (CD68) in PMA-activated THP1 cells induced by IFN-γ and LPS in the presence or absence of S100A7 protein, and our analysis showed a significant decrease of CD68 expression in macrophages treated with additional S100A7 protein (Fig. 1f). The above results suggested that S100A7 inhibited M1 macrophage polarization. Interestingly, numerous studies have identified CD68^+^ macrophages as a major source of CXCL9.^3,4^

**Fig. 1:**
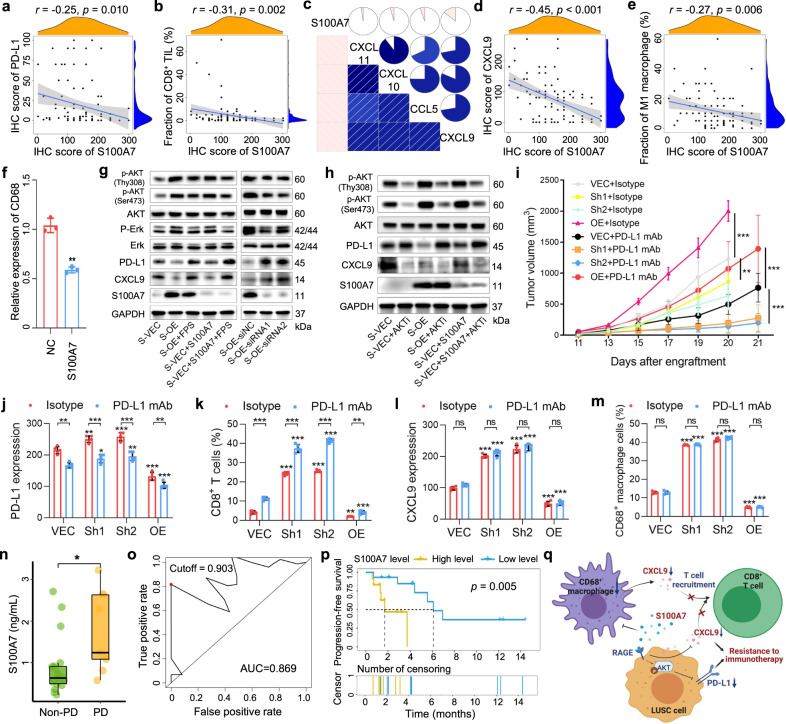
The impact of S100A7 in TIME characteristics and efficacy of immunotherapy in LUSC. **a**, **b**, **d**, and **e** IHC analysis of S100A7 and **a** PD-L1 expression, **b** CD8^+^ TIL infiltration, **d** CXCL9 expression, and **e** M1 macrophage infiltration in tumor specimens of patients with LUSC. **c** Cross-correlogram based on Pearson’s *r* values among the expression of S100A7, CXCL9, CXCL10, and CXCL11 in LUSC samples from the TCGA dataset. **f** CD68 expression of PMA-activated THP1 cells induced by IFN-γ and LPS in the presence or absence of S100A7 protein. NC group: PMA + IFN-γ + LPS; S100A7 group: PMA + IFN-γ + LPS + S100A7. **g** and **h** Western blot analysis of S100A7, PD-L1, and CXCL9 expression, and kinase signaling pathways in H226 cells with different treatments. GAPDH expression was used as a loading control. S-VEC: control H226 cells; S-OE: S100A7-overexpressing H226 cells. FPS: RAGE-specific inhibitor (FPS-ZM1); S100A7: recombinant human S100A7 protein; AKTi: AKT-specific inhibitor (MK-2206 2HCL). **i** The mice bearing control (VEC), S100A7a-knockdown (Sh1 and Sh2), and S100A7a-overexpression (OE) KLN205 cells (*n* = 5) were treated with either the isotype control or anti-PD-L1 monoclonal antibody (anti-PD-L1 mAb). The mice treated with isotype control were sacrificed by carbon dioxide asphyxiation to harvest tumors for IHC analysis on day 20 because the tumor nodules were too large. **j**–**m** IHC analysis of **j** PD-L1 expression, **k** CD8^+^ T cells infiltration, **l** CXCL9 expression, and **m** CD68^+^ macrophage infiltration in each treated group. **n** The distribution of baseline plasma S100A7 levels between the patients with progressive disease (PD) and non-PD. **o** ROC analysis of baseline plasma S100A7 levels for progression-free survival (PFS) of patients with LUSC receiving anti-PD-1 immunotherapy. **p** Kaplan–Meier survival curve of PFS of patients with LUSC receiving anti-PD-1 immunotherapy based on baseline plasma S100A7 levels. **q** The graphical abstract of the study. The graphical abstract was created with BioRender.com. ns: *p* > 0.05; *p < 0.05^;^ **p < 0.01; ***p < 0.001

Given that CXCL9 was also found in tumor cells (Supplementary Fig. S2), we measured the CXCL9 expression of control and S100A7-overexpressing LUSC cells using RT-qPCR and western blotting. The results revealed that S100A7, whether endogenously produced or exogenously added in H226 cells, decreased CXCL9 expression, whereas S100A7 silencing or treatment with the receptor for advanced glycation end products (RAGE) inhibitor (FPS-ZM1) increased CXCL9 expression (Fig. 1g and Supplementary Fig. S3f, g). Further pathway study indicated that CXCL9 expression was slightly attenuated via AKT inhibitor (Fig. 1h and Supplementary Fig. S3h). Previous studies have demonstrated that S100A7 can exert its functions and promote the activation of downstream signal pathways through the RAGE.^1,2^ Consistently, in our study, overexpression or exogenous introduction of S100A7 protein activated the p-Erk and p-AKT pathways (Fig. 1g). Moreover, we also observed that the expression level of PD-L1was obviously deregulated by S100A7 (Fig. 1g and Supplementary Fig. S3c). Previous studies have reported that activating the p-Erk signaling pathway increases PD-L1 expression.^5^ Notably, our study demonstrated that PD-L1 expression could be partly abrogated by either S100A7 knockdown or treatment with the RAGE or AKT inhibitor (Fig. 1g, h and Supplementary Fig. S3c–e). Based on these findings, we speculated that S100A7 overexpression not only decreased PD-L1 level via the activation of the p-AKT pathway but also suppressed the secretion of chemokine CXCL9 in tumor microenvironment by down-regulating the infiltration of CD68^+^ macrophages and CXCL9 level in the tumor cell. These mechanisms further down-regulated the infiltration of CD8^+^ TILs, which in turn built up an immunosuppressive TIME that was primarily resistant to ICIs.

Considering the relationship between S100A7 expression and an immunosuppressive TIME, we wanted to determine the effect of dysregulating S100A7 on the immunotherapy response observed in LUSC. For this, we used a murine model bearing the mouse homolog of human S100A7, S100A7a, in subsequent experiments (Supplementary Figs. S4 and S5a). Compared to the vector control, the anti-tumor effects of anti-PD-L1 inhibitors were stronger in the S100A7a-knockdown group (*p* < 0.001; Fig. 1i and supplementary Fig. S5c). Meanwhile, in line with our previous results, knocking down S100A7a decreased the growth ability of tumor nodules in the absence of anti-PD-L1 inhibitors (*p* < 0.001; Fig. 1i and Supplementary Fig. S5b). Immunohistochemistry studies revealed a higher PD-L1 and CXCL9 expression and increased CD8^+^ T cells and CD68^+^ macrophage infiltration in mouse tumor specimens with S100A7a-knockdown (Fig. 1j–m). More importantly, administration of anti-PD-L1 drugs in S100A7a-overexpressing mice did not elicit a more remarkable change in PD-L1 expression and CD8^+^ T cell infiltration compared to other groups (Fig. 1j, k).

Based on previous reports that S100A7 can be secreted outside the cell and detected in plasma,^2^ a cohort of 27 LUSC patients treated with ICIs was enrolled to investigate the association between baseline plasma levels of S100A7 and the efficacy of ICIs (Supplementary Table S1). Higher baseline plasma levels of S100A7 were observed in LUSC patients with progressive disease (*p* = 0.039; Fig. 1n). Furthermore, the area under the curve of the receiver operating characteristic (ROC) curve was calculated to analyze the predictive power of S100A7 level in baseline plasma for immunotherapy response, and the value was 0.869 (Fig. 1o). We then assigned patients into two groups based on the best cutoff values calculated from ROC curve (cutoff = 0.903; Fig. 1o). In our analyses, higher baseline plasma levels of S100A7 were found to be significantly related to worse progression-free survival rates (*p* = 0.005; Fig. 1p). In addition, univariate and multivariate Cox regression analyses revealed that baseline plasma levels of S100A7 were an independent prognostic factor (Supplementary Table S3).

Taken together, we demonstrated that the upregulation of S100A7 could result in ICIs resistance due to a remodeled immunosuppressive TIME characterized by a decreased PD-L1 expression via the activation of the p-AKT pathway, and decreased CD8^+^ TILs infiltration via the downregulation of CXCL9 expression (Fig. 1q). We also confirmed that targeted inhibition of S100A7 may strengthen the efficacy of ICIs via the generation of an inflammatory phenotype, however, lack of anti-S100A7 inhibitors limited our clinical application. Lastly, we identified the predictive power of the S100A7 level in baseline plasma, which could further guide the use of ICIs for LUSC patients. However, the limitations of the small sample size should be acknowledged, thus further validation of this biomarker in future large-scale studies is warranted.

## Supplementary information

Supplementary_Materials

## Data Availability

The datasets used and/or analyzed in this study can be obtained from the corresponding authors as reasonably required.

## References

[1] Lu Z (2018). The TGFbeta-induced lncRNA TBILA promotes non-small cell lung cancer progression in vitro and in vivo via cis-regulating HGAL and activating S100A7/JAB1 signaling. Cancer Lett..

[2] Lu Z (2021). S100A7 as a potential diagnostic and prognostic biomarker of esophageal squamous cell carcinoma promotes M2 macrophage infiltration and angiogenesis. Clin. Transl. Med..

[3] House IG (2020). Macrophage-derived CXCL9 and CXCL10 are required for antitumor immune responses following immune checkpoint blockade. Clin. Cancer Res..

[4] Tokunaga R (2018). CXCL9, CXCL10, CXCL11/CXCR3 axis for immune activation—a target for novel cancer therapy. Cancer Treat. Rev..

[5] Coelho MA (2017). Oncogenic RAS signaling promotes tumor immunoresistance by stabilizing PD-L1 mRNA. Immunity.

