# Supplementary material for: S100A7 attenuates immunotherapy by enhancing immunosuppressive tumor microenvironment in lung squamous cell carcinoma
Source: Signal Transduct Target Ther. 2022 Oct 21;7:368. doi: 10.1038/s41392-022-01196-4 (PMC9584899; doi:10.1038/s41392-022-01196-4)
Supplement: Supplementary file 1 — Supplementary_Materials [file 41392_2022_1196_MOESM1_ESM.docx]

Supplementary Materials for

S100A7 attenuates immunotherapy by enhancing immunosuppressive tumor microenvironment in lung squamous cell carcinoma

Chengming Liu, Sufei Zheng, Zhiliang Lu, Zhanyu Wang, Sihui Wang, Xiaoli Feng, Yan Wang, Nan Sun, Jie He

Correspondence to: [prof.jiehe@gmail.com](mailto:prof.jiehe@gmail.com) and [sunnan@cicams.ac.cn](mailto:sunnan@cicams.ac.cn)

**This PDF file includes:**

Materials and Methods

Figures. S1 to S5

Tables S1 to S3

Materials and Methods

**Tumor specimens and immunohistochemistry (IHC)**

Tumor samples were resected from 102 patients with lung squamous carcinoma (LUSC) between January 2013 and December 2017 in Cancer Hospital and Institute, Chinese Academy of Medical Sciences (CICAMS, Beijing, China) from January 2008 to December 2013. None of the patients had received radiotherapy or chemotherapy before surgery, nor were any of them diagnosed with other cancers within 3 years before surgery. These specimens were analyzed for S100A7 expression, PD-L1 expression, CXCL9 expression, and numbers of CD8^+^ T cells and CD68^+^ macrophages. Using IHC, tumor sections were stained for S100A7 (anti-human S100A7 monoclonal antibody, Cat# ab13680; Abcam, UK), PD-L1 (pre-diluted anti-human PD-L1 monoclonal antibody, clone SP263, Cat# 740-4907; Ventana, USA) using an automated Ventana Benchmark XT instrument, CXCL9 (anti-human CXCL9 polyclonal antibody, Cat# 22355-1-AP; Proteintech, USA), CD8 (pre-diluted anti-human CD8 monoclonal antibody, Cat# ZA-0508; Zsbio Tech, China) and CD68 (anti-human CD68 monoclonal antibody, Cat# ab213363; Abcam, UK).

The staining score of S100A7 and CXCL9 in every sample was calculated using the following formula: staining score = staining intensity × % of positive tumor cells × 100. Scoring based on the staining intensity: no color development was rated as 0 (negative), and color development intensity was pale yellow as 1 (weak), yellow as 2 (moderate), and brown-yellow as 3 (strong). Ten fields were randomly chosen under a high-power microscope (×400). Ten fields were randomly selected under a high-power microscope (×400) in subcutaneous tumor nodule of per mouse. The average value was taken to calculate the percentage of immune cells that stain positively compared to all immune cells in view. All slides were assessed by two experienced pathologists blinded to the clinical parameters. In cases of disagreement, the slides were reviewed by both observers together to achieve consensus^1^. The PD-L1 tumor proportion score and the proportion of immune cells were assessed according to the evaluation criteria of the previously published approach^2^.

**Intratumoral immune cell composition analysis**

CIBERSORT (https://cibersort.stanford.edu/) was used to calculate immune cell type fractions with the gene expression profile of each patient at 1000 permutations and the results was further filtered using *p*-value < 0.05. Quantile normalization was used to remove confounding effects.

**Cell lines and reagents**

Human H226 cells and THP-1 cells were cultured in RPMI-1640 media (Cat# 10-040-CV, Corning, USA) containing 10% FBS and an antibiotic mixture (100 U/mL penicillin and 100 µg/mL streptomycin, Cat# 15140-122, Gibco, USA). HEK-293T cells were cultured in DMEM media (Cat# 10-013-CVR, Corning, USA) containing 10% FBS and an antibiotic mixture. Mouse KLN205 cells (clones from the lungs of a DBA-2J mouse with LUSC) were cultured in DMEM media containing 10% FBS and an antibiotic mixture.

The cytokine used in this study included human recombinant S100A7 protein (Cat# 9085-SA, R&D Systems, USA, 100ng/ml) and human recombinant IFN-γ protein (Cat# CAA31639, R&D Systems, USA, 20ng/ml). The inhibitors used in this study included RAGE inhibitor (FPS-ZM1-Calbiochem, Cat# 553030, Sigma-Aldrich, USA) and AKT-specific inhibitor (MK-2206 2HCL, Cat# S1078, Selleck, China). The specific siRNAs of S100A7 and non-targeting control siRNA were purchased from Thermo Fisher (Cat# s12420, s12421 and 13778030).

**THP-1 macrophage differentiation and polarization**

THP-1 cells were plated on surfaces at 1x10^5^ cells/cm^2^ in RPMI-1640 with 10% FBS plus 100ng/mL PMA (Cat# 16561-29-8, Sigma-Aldrich, USA) for 72 hours to differentiate into M0-macrophages. M1 polarization was induced by 20 ng/mL IFN-γ and 20 ng/mL LPS (Cat#L2880, Sigma-Aldrich, USA) for another 4 days.

**Lentiviral human S100A7 stable overexpression**

For stable overexpression of exogenous human S100A7, full-length S100A7 cDNA was ligated into the pCDH-CMV-MCS-EF1-Puro (CD510B) vector. To produce lentivirus containing the human S100A7 gene, HEK-293T cells were co-transfected with the S100A7 vector and Invitrogen™ ViraPower™ Lentiviral Packaging Mix (pLP1, pLP2, and pLP/VSVG, Cat# K497500, Invitrogen, USA) using Lipofectamine 3000 (Cat# L3000015, Invitrogen, USA) strictly according to the manufacturer’s guidelines. Infectious lentiviruses were harvested at 48 hours after transfection and filtered through Amicon Ultra-15 Centrifugal Filter Devices (Cat# UFC910096, Millipore, Massachusetts, USA). H226 cells were then infected with harvested lentiviruses and exposed to 5 μg/mL polybrene (Cat# P4505, Sigma-Aldrich, USA) and selected with 2 μg/mL puromycin (Cat# A1113803, Gibco, USA). The levels of S100A7 in the infected cells were confirmed by RT-qPCR and western blot 96 hours after puromycin selection.

**Lentiviral mouse S100A7a stable overexpression and knock-down**

For stable overexpression of exogenous mouse S100A7a, full-length cDNA was ligated into the plasmid vector (pLV-EF1a-hluc-P2A-mNeongreen-CMV-3Xflag-P2A-puro). Short hairpin RNAs (shRNAs) were applied to construct S100A7a knock-down cell lines, and sequence of shRNAs targeted S100A7a were listed (Sh1:5’-GGCAGTCTCTCATCACCAGAA-3’; Sh2: 5’-GCCTGATCATGAACAGTGTAC-3’; Syngentech Co., Ltd., Beijing). Then, the recombinant plasmids and packaging plasmids were co-transfected into HEK-293T cells to obtain infectious lentivirus particles using Lipofectamine 3000. The lentivirus-containing supernatant was collected at 48 hours and puriﬁed with Amicon Ultra-15 Centrifugal Filter Devices. The puriﬁed lentivirus was supplemented with 5 μg/mL polybrene and used to infect the KLN205 cells. We used corresponding empty vectors as controls. Stable cell lines were obtained after 14 days of selection with puromycin.

**RNA extraction, RT-qPCR, western blot, and flow cytometry**

RNA extraction, RT-qPCR, western blot, and flow cytometry were performed as previous study^3^. Total RNA was obtained with TRIzol RNA-Extraction reagent according to the instructions. First-Strand cDNA Synthesis kit (Cat# K1612, Life Technologies, California, USA) was used for reverse transcription. RT-qPCR was performed with SYBR™ Select Master Mix (Cat# 4472908, Life Technologies, California, USA) on ABI 7900HT Real-Time PCR thermocycler (Life Technologies, California, USA). The PCR primers used in this study are shown in **supplementary Table S2**. The antibodies for western blot used in this study are listed as follows: S100A7 (Cat# ab13680; Abcam, UK), PD-L1 (Cat# 13684T, CST, Danvers, MA, USA), CXCL9 (Cat# 22355-1-AP; Proteintech, USA), Erk (Cat# 4695, CST, Danvers, MA, USA), Phospho-ERK (Thr202/Tyr204) (Cat# 4370, CST, Danvers, MA, USA), AKT (Cat# 4691, CST, Danvers, MA, USA), Phospho-AKT (Thr308) (Cat# 13038, CST, Danvers, MA, USA) and GAPDH (Cat# ab8245, Abcam, Cambridge, UK), Phospho-AKT (Ser473) (Cat# 4060, CST, Danvers, MA, USA).

***In vivo* mouse experiments**

Male DBA-2J mice (SPF, 5–6 weeks old) were purchased from Huafukang Bioscience Co., Ltd. (Beijing, China). The animals were fed a standard commercial diet produced by the Experimental Animal Center of the CICAMS and were maintained in specific pathogen-free conditions under a 12-h light-dark schedule. The temperature and humidity of the animal house was maintained at 26 °C–28 °C and 60 ± 5% respectively. All procedures were approved by the Animal Care and Use Committee of CICAMS. As shown in **supplementary Fig. S5a**, to establish the mouse model of LUSC, DBA-2J mice were subcutaneously implanted with murine KLN205-derived xenografts, respectively. Notably, the KLN205 cells were stably transfected with empty vector (VEC), S100A7a-overexpression vector (OE), and S100A7a-knockown vector (Sh1 and Sh2). We measured tumor size using a caliper every three days and calculated the tumor volume based on the formula V = L×W^2^/2 (V: tumor volume; L: tumor length; W: tumor width). The mice were randomly placed into five groups with five mice/group and given different treatments when the maximum tumor diameter reached 6 mm. The treatment regimens involved isotype control (10 mg/kg three times a week, Cat#BE0090, Bio X Cell, USA) and anti-PD-L1 mAb (10 mg/kg three times a week, Cat#BE0101, Bio X Cell, USA) as described in **supplementary Fig. S5a**. The endpoints were defined when the maximum tumor diameter reached 20 mm, the weight loss was greater than 2 g, or death. All mice were sacrificed by carbon dioxide asphyxiation to harvest tumors. Notably, the mice treated with isotype control were sacrificed by carbon dioxide asphyxiation to harvest tumors for IHC analysis on day 20 because the maximum tumor diameter reached 20 mm.

Harvested tumors were fixed in formalin, embedded in paraffin, and sectioned (4 µm). Using IHC, tumor sections were stained for PD-L1 (anti-mouse PD-L1 antibody, Cat# ab238697; Abcam, UK), CD8 (anti-mouse CD8 antibody, Cat# 98941; CST, USA), CXCL9 ((anti-mouse CXCL9 polyclonal antibody, Cat# 22355-1-AP; Proteintech, USA) and CD68 (anti-mouse CD68 antibody, Cat# GB113109; Servicebio, China).

**Clinical cohorts and response evaluation**

A cohort of 27 patients diagnosed with LUSC and treated with anti-PD-1 inhibitors from January 2018 to December 2019 at the CICAMS was included in the present study. Enrolled patients received immunotherapy once every three weeks. Radiologic assessments, including computed tomography, were conducted once every six weeks. If necessary, head magnetic resonance imaging was simultaneously performed. The tumor response was assessed according to the Response Evaluation Criteria in Solid Tumors (version 1.1) and was categorized as complete response (CR), partial response (PR), stable disease (SD), or progressive disease (PD). Progression-free survival (PFS) and OS were defined as the time from the initiation of ICIs administration to the time of PD and from the beginning of ICIs to death, respectively. The last follow-up assessment was performed on January 2, 2021. The Ethics Committee of CICAMS approved this study (approval number 20/242-2438). Clinical characteristics of these LUSC patients are described in **supplementary Table S1**.

**Sample collection and enzyme-linked immunosorbent assay (ELISA)**

Blood samples were collected in an EDTA tube prior to the administration of anti-PD-1 immunotherapy. All samples were processed within 2 hours of collection, and centrifuged at 3,000 rpm for 10 min at 4 ℃. The upper plasma fraction was stored at −80 °C until assayed. Plasma samples were obtained from LUSC patients to perform the ELISA. The human CircuLex S100A7/Psoriasin ELISA Kit (Cat# CY-8073, MBL, Japan) was applied to measure the plasma levels of S100A7 according with the instructions. Three biological duplicates were designed for each sample. The optical densities (OD) were measured immediately after adding stop solution at 450 nm using a spectrophotometer (SpectraMax® 190, Molecular Devices, Sunnyvale, CA, USA).

**Statistical analysis**

Data analysis was conducted using R software (version 3.6.0) and GraphPad Prism software (version 8.0, Graph Pad, San Diego, CA, USA). Differences between independent variables were evaluated using the Kruskal-Wallis H test or Mann-Whitney U test. Fisher’s exact test was used to analyze categorical variables. Correlation coefficients were obtained using Pearson correlation analysis. Survival was assessed with the log-rank test and Kaplan-Meier analysis. Furthermore, Cox regression was conducted for univariate and multivariate analyses of prognosis. All statistical analyses were double-sided, and statistical significance was considered as *p* values less than 0.05.

Supplementary References

1 Liu, C. et al. Development and external validation of a composite immune-clinical prognostic model associated with EGFR mutation in East-Asian patients with lung adenocarcinoma. *Ther Adv Med Oncol.* **13**, 17588359211006949 (2021).

2 Liu, C. et al. The superior efficacy of anti-PD-1/PD-L1 immunotherapy in KRAS-mutant non-small cell lung cancer that correlates with an inflammatory phenotype and increased immunogenicity. *Cancer Lett.* **470**, 95-105 (2020).

3 Lei, Y. et al. The membrane-bound and soluble form of melanotransferrin function independently in the diagnosis and targeted therapy of lung cancer. *Cell Death Dis.* **11**, 933 (2020).


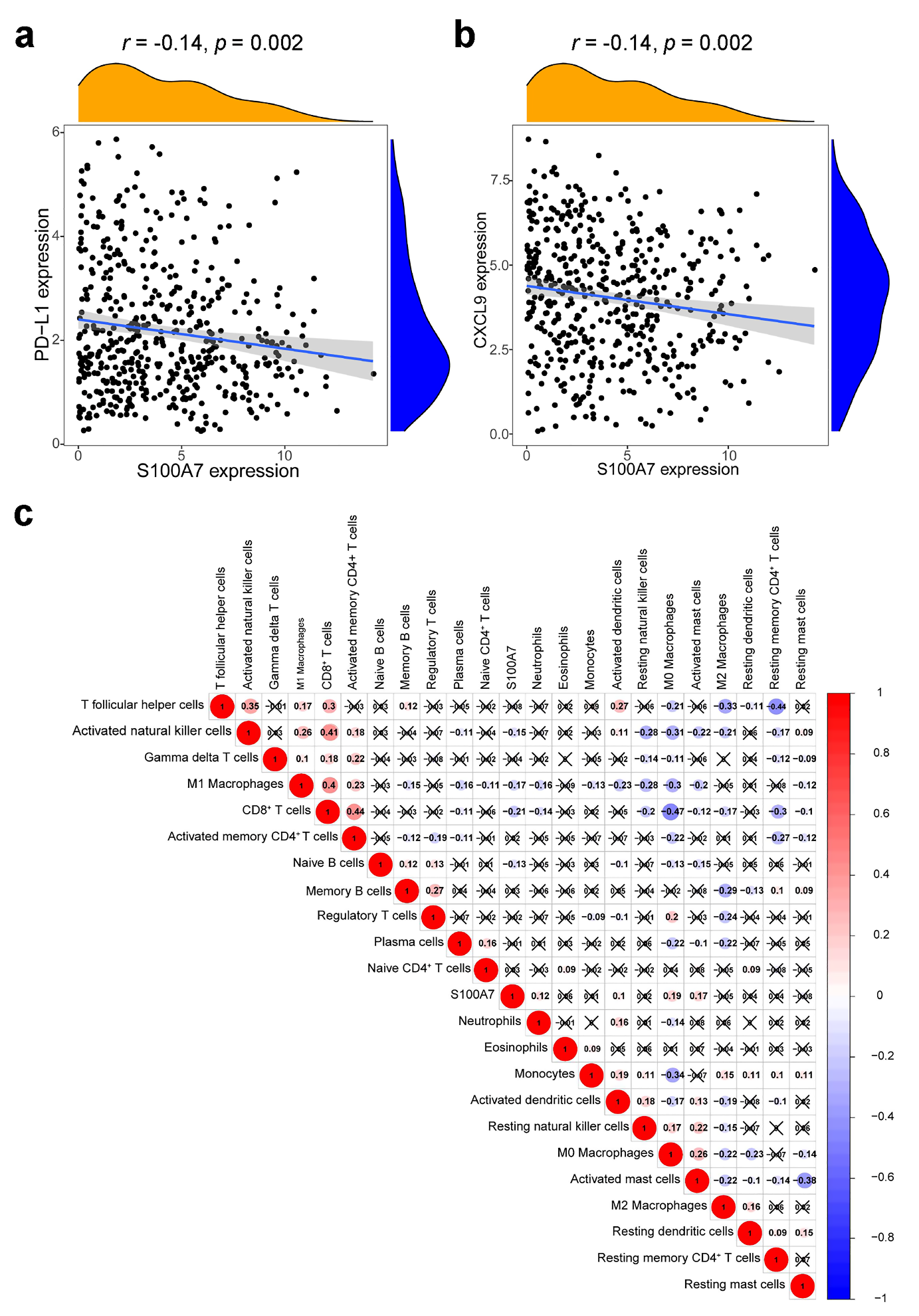


Figure. S1.

Relationships of S100A7 with TIME in LUSC.

**(a and b)** The relationships of S100A7 expression with **(a)** PD-L1 expression and **(b)** CXCL9 expression in LUSC samples of TCGA database. **(c)** Cross-correlogram based on Pearson’s *r* values among S100A7 expression and 22 tumor-infiltrated immune cells in LUSC samples via the CIBERSORT.


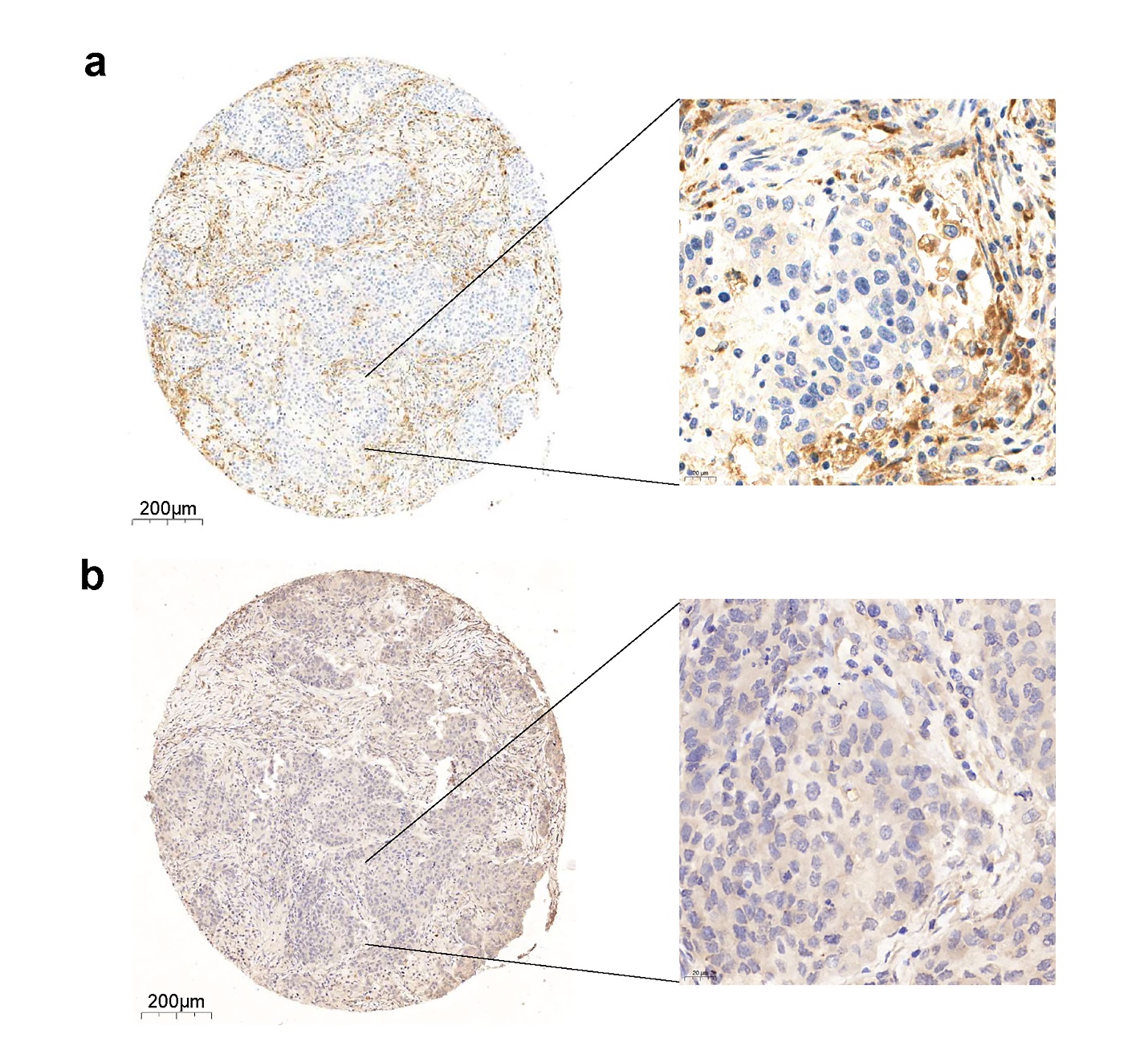


Figure. S2.

Representative figures of IHC.

Representative figures of **(a)** CD68^+^ macrophages and **(b)** CXCL9 expression. Positive staining of CXCL9 protein can be observed in CD68^+^ macrophages, which mainly be positively stained in tumor stroma.


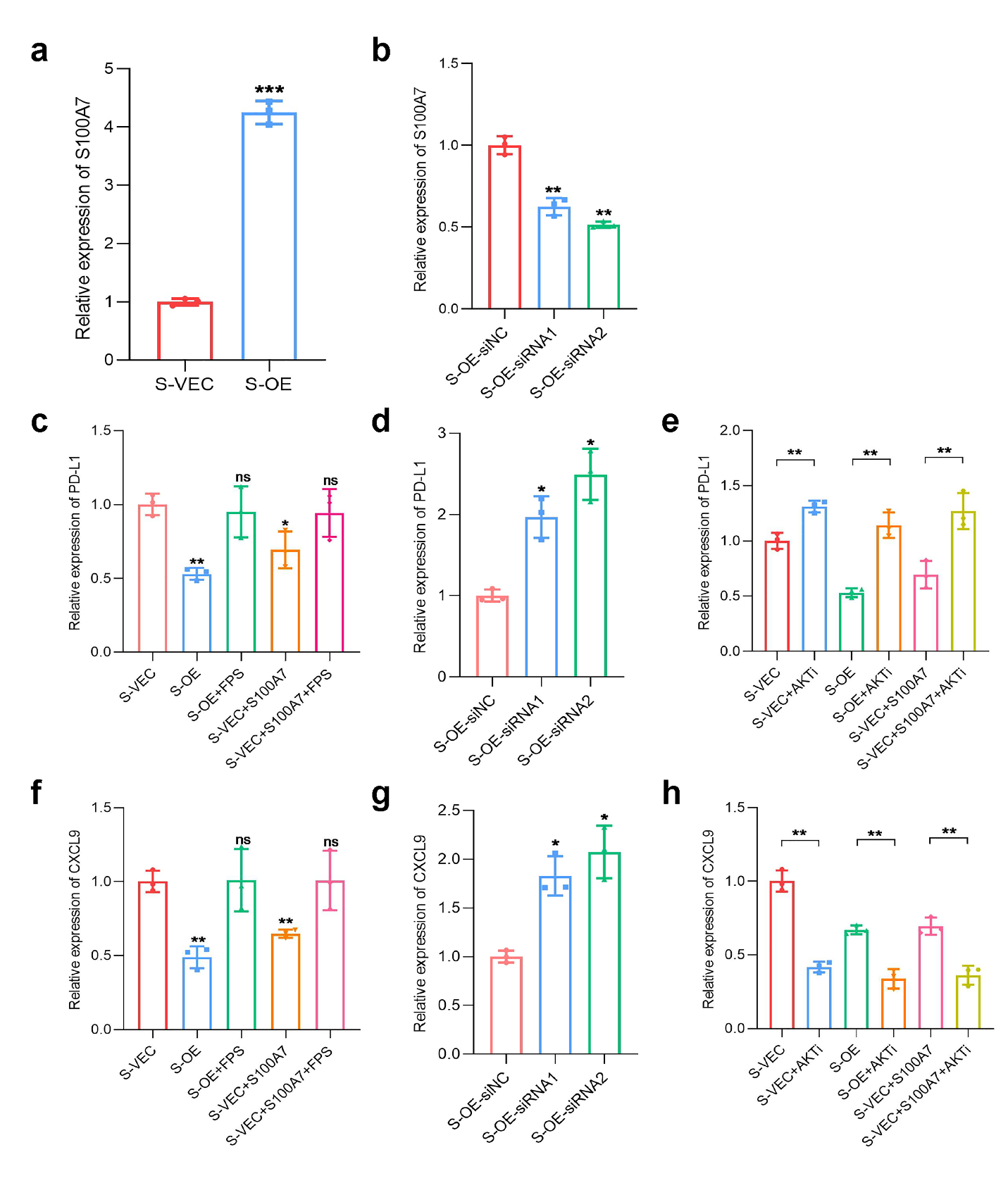


Figure. S3.

S100A7, PD-L1 and CXCL9 expression of H226 cells with different interventions.

RT-qPCR analysis of **(a-b)** S100A7 expression, **(c-e)** PD-L1 expression and **(f-h)** CXCL9 expression in H226 cells with different interventions. S-VEC: control H226 cells; S-OE: S100A7-overexpressing H226 cells. FPS: RAGE receptor inhibitor (FPS-ZM1); S100A7: recombinant human S100A7 protein; AKTi: AKT-specific inhibitor (MK-2206 2HCL). ns: *p* > 0.05; *: *p* < 0.05; **: *p* < 0.01; ***: *p* < 0.001.


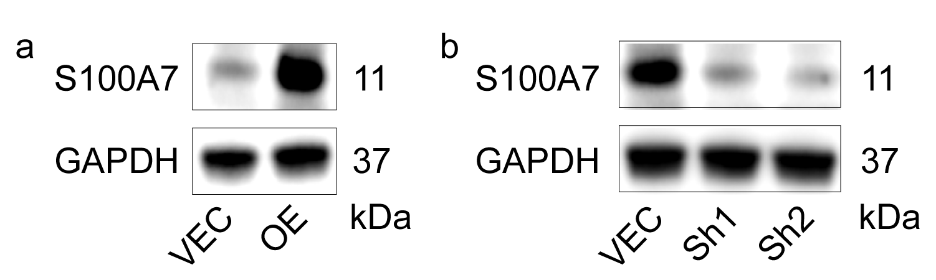


Figure. S4.

Western blotting analysis of S100A7a expression in KLN205 cells stably transfected with empty vector (VEC), S100A7a-overexpressing vector (OE), and S100A7a-knockdown vector (Sh1 and Sh2).


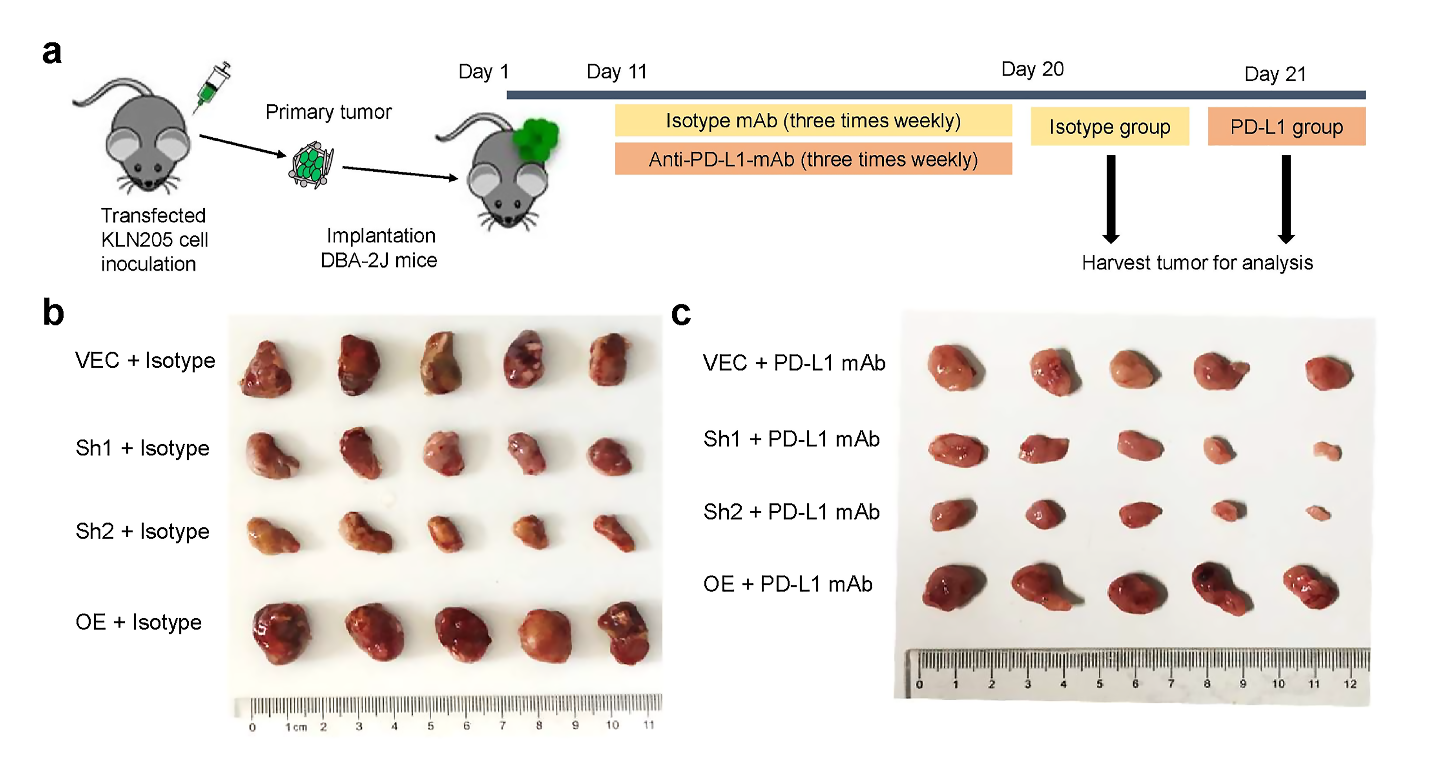


Figure. S5.

The association between dysregulation of S100A7 and immunotherapeutic response in a murine model of LUSC.

1. Schemas for constructing the murine model of LUSC and dosing schedule. **(b and c)** Representative illustrations of tumor nodules in the treatment of **(b)** isotype control or **(c)** anti-PD-L1 inhibitor.

Table S1.

Demographic characteristics of LUSC patients treated with anti-PD-1 immunotherapy in the CICAMS cohort.

| Characteristics | Whole Population |
| --- | --- |
| Case No.  Gender, *n* (%) | 27 |
| Male | 23 (85.2) |
| Female | 4 (14.8) |
| Age, *n* (%) |  |
| ＜60 | 11 (40.7) |
| ≥60 | 16 (59.3) |
| Smoking history, *n* (%) |  |
| Never | 6 (22.2) |
| Ever | 21 (77.8） |
| Staging, *n* (%) |  |
| III | 6 (22.2) |
| IV | 21 (77.8） |
| ECOG PS, *n* (%) |  |
| 0 | 1 (3.7） |
| 1 | 21 (77.8) |
| 2 | 5 (18.5) |
| Therapy lines, *n* (%) |  |
| 1 | 6 (22.2） |
| 2 | 13 (48.2) |
| ≥3 | 8 (29.6) |
| Study drugs, *n* (%) |  |
| Pembrolizumab | 15 (55.6) |
| Sintilimab | 12(44.4) |
| Response evaluation, *n* (%) |  |
| PR | 4 (14.8） |
| SD | 14 (51.9） |
| PD | 9 (33.3) |

Table S2.

Primers used in this study.

| **Genes** |  | **Primer Sequences** | **Length** |
| --- | --- | --- | --- |
| GAPDH | Forward | CCTGGTATGACAACGAATTTG | 24 |
|  | Reverse | CAGTGAGGGTCTCTCTCTTCC | 24 |
| S100A7 | Forward | AACTTCCTTAGTGCCTGTG | 19 |
|  | Reverse | TGGTAGTCTGTGGCTATGTC | 20 |
| PD-L1 | Forward | CCATCTTATTATGCCTTGGTGTAG | 24 |
|  | Reverse | TTTGCTTCTTTGAGTTTGTATCTTG | 25 |
| CXCL9 | Forward | ACCACATCCCACTCACAAC | 19 |
|  | Reverse | GGCTTAGGACTTGCTGACAT | 20 |
| CD68 | Forward | CGAGCATCATTCTTTCACCAGCT | 22 |
|  | Reverse | ATGAGAGGCAGCAAGATGGACC | 22 |

Table S3.

Univariate and multivariate regression analyses of the relationship between baseline plasma S100A7 levels and clinical factors for the prediction of PFS.

|  | Univariable analysis | | Multivariable analysis | |
| --- | --- | --- | --- | --- |
|  | *p* value | HR（95%CI） | *p* value | HR（95%CI） |
| Gender | 0.708 | 1.494（0.183-12.194） | 0.326 | 2.483（0.076-80.638） |
| Age | 0.732 | 1.012（0.944-1.086） | 0.609 | 1.036（0.965-1.113） |
| Smoking history | 0.631 | 1.453（0.317-6.666） | 0.987 | 1.020（0.096-10.872） |
| Staging | 0.704 | 0.776（0.209-2.881） | 0.660 | 0.722（0.169-3.085） |
| ECOG PS | 0.366 | 0.565（0.164-1.950） | 0.578 | 0.604（0.102-3.574） |
| Therapy lines | 0.457 | 1.361（0.605-3.061） | 0.178 | 2.152（0.705-6.568） |
| Study drugs | 0.616 | 0.745（0.235-2.357） | 0.289 | 0.463（0.112-1.918） |
| **S100A7 level** | **0.009** | 2.731（1.284-5.811） | **0.008** | 3.328（1.374-8.063） |
